# Large language models as data-driven engines for benchmarking preventive and clinical knowledge in Chinese dental examinations

**DOI:** 10.3389/froh.2026.1849721

**Published:** 2026-06-19

**Authors:** Yong Zeng, Xinyi Hu, Wei Liu, Ke Deng, Meiqin Zhou, Yao Wang, Ling Ma, Qi Liu, He Meng

**Affiliations:** 1 School of Dentistry, Shenzhen University Medical School, Shenzhen, Guangdong, China; 2 Department of Stomatology, Shenzhen University General Hospital, Shenzhen University, Shenzhen, Guangdong, China; 3 Institute of Stomatological Research, Shenzhen University, Shenzhen, Guangdong, China; 4 Division of Periodontology and Implant Dentistry, The Faculty of Dentistry, The University of Hong Kong, Hong Kong SAR, China; 5 International Cancer Center, Shenzhen University, Shenzhen, Guangdong, China

**Keywords:** artificial intelligence in dentistry, data-driven technologies, dental education, educational decision-support, examination performance benchmarking, large language models

## Abstract

**Introduction:**

Digital and data–driven technologies are increasingly shaping preventive oral healthcare and dental education. While prior studies have explored large language model (LLM) performance in non–English examination settings, their role in benchmarking standardized clinical knowledge against real student performance under identical institutional conditions remains insufficiently examined.

**Methods:**

This study evaluated GPT–4o, GPT–4.5, and DeepSeek-R1 using 300 standardized dental competency items from institutional examinations (2023–2025). Model performance was benchmarked against data from a real student cohort who completed the same examinations, and intra–model consistency was assessed across multiple independent trials.

**Results:**

DeepSeek–R1 achieved the highest overall accuracy (92.33%), significantly outperforming GPT–4.5 and GPT–4o in 2023 and 2024, while showing comparable performance to GPT–4.5 in 2025. Model type was the predominant factor associated with accuracy differences, whereas knowledge domain did not significantly affect results. DeepSeek–R1 and GPT–4.5 also demonstrated greater response consistency than GPT–4o.

**Discussion:**

These findings suggest that optimized LLMs demonstrate strong alignment with standardized examination criteria and student performance patterns, supporting their potential role as supplementary tools for curriculum evaluation in preventive dental education. Caution is warranted in generalizing these results beyond the single-institutional, text-only, Mandarin-language context.

## 1 Introduction

Large language models have evolved rapidly from early rule-based natural language processing systems to contemporary transformer-based architectures capable of generating coherent, contextually relevant text. The introduction of models such as GPT-3 marked a turning point in generative AI, demonstrating emergent reasoning abilities across diverse domains. Subsequent iterations—including GPT-4 and its variants—have incorporated reinforcement learning from human feedback (RLHF) and multimodal training, further enhancing performance on specialized knowledge tasks (1, 2). More recently, open-source models such as DeepSeek-R1 have leveraged large-scale reinforcement learning to achieve competitive reasoning performance, expanding access to advanced AI capabilities (3). These developments have prompted growing interest in evaluating LLM performance in high-stakes professional and educational contexts, including medical and dental examinations (4, 5).

Within dental education specifically, these advances are reshaping clinical training, personalized education, and data-informed decision-making (5). Compared with traditional rule-based AI systems, modern LLMs exhibit superior natural language understanding, generation, and dialogic interaction. These capabilities suggest potential applications in areas such as curriculum support, clinical reasoning training, and examination performance assessment (1, 2, 6, 7). Empirical studies have demonstrated that LLMs can achieve passing-level reasoning in licensing-style assessments across multiple cultural contexts (4, 8).

Recent studies in dental education have begun to explore the use of LLMs and other natural language processing (NLP)-based tools to support various aspects of teaching and clinical practice. Reported applications include improvements in diagnostic documentation accuracy, clinical communication efficiency, and the delivery of evidence-based educational content (9). Several studies have also evaluated LLM reasoning using standardized clinical assessment items across specialties and national licensing examinations in different countries (10–19). These studies demonstrate that advanced models can perform competently on structured examination tasks in diverse linguistic and educational settings. Recent scoping reviews have further systematized this landscape: El-Hakim et al. (20) categorized AI applications into four domains while emphasizing ethical and variability challenges, and a comprehensive review identified seven LLM application areas with dental examinations (*n* = 24) representing the largest category, alongside persistent concerns regarding information accuracy and cautious implementation (21).

However, most existing evaluations assess LLM performance primarily against answer keys or predefined passing thresholds, and rarely situate model outputs within an authentic educational measurement context by directly comparing them with the performance of real student cohorts under identical examination conditions. This gap is particularly relevant for Chinese-language dental education, where localized benchmarking data remain limited despite growing interest in integrating AI into educational systems (6, 16, 18). Whether LLM performance aligns with actual student-level competency patterns in institutional examinations therefore remains unclear.

In this study, we evaluated the accuracy and response consistency of three state-of-the-art LLMs (GPT-4o, GPT-4.5, and DeepSeek-R1) using 300 standardized competency assessment items derived from institutional dental examinations between 2023 and 2025. These items span foundational science and clinical reasoning domains relevant to preventive and diagnostic oral healthcare. Model performance was benchmarked against data from a real student cohort who completed the same examinations, and intra-model consistency was assessed across multiple independent runs.

By embedding LLM evaluation within a real-world educational assessment framework, this study provides empirical evidence on the examination-answering performance of optimized LLMs and their alignment with standardized curricular knowledge and real student performance patterns in dental education (1, 6, 12, 22).

## 2 Materials and methods

### 2.1 Large language models

To evaluate the potential of digital technologies as engines for clinical knowledge benchmarking, three state-of-the-art LLMs were selected: GPT-4o and GPT-4.5 (OpenAI, San Francisco, CA, USA), and DeepSeek-R1 (DeepSeek-AI, Hangzhou, China). GPT-4o (OpenAI, San Francisco, CA, USA) was included as a widely-used commercial baseline given its documented performance in prior medical and dental benchmarking studies (4, 6, 13, 14). GPT-4.5 (OpenAI) was selected as a successor model to GPT-4o to assess whether incremental architectural updates translate to measurable gains in specialized domain performance. DeepSeek-R1 (DeepSeek-AI, Hangzhou, China) was specifically included because (a) its training emphasized multilingual and Chinese-language corpora, making it particularly relevant for Chinese dental education contexts (3); (b) its open-weight architecture facilitates reproducibility and local deployment; and (c) recent benchmarking studies have reported strong performance on medical reasoning tasks (3, 18). Together, these models represent a spectrum of contemporary LLM architectures (commercial closed-source and open-weight, generalist and reasoning-optimized), enabling comparison across design philosophies while maintaining relevance to dental education applications.

### 2.2 Standardized knowledge dataset and classification

A standardized dataset consisting of 300 single-best-answer competency assessment items was derived from institutional dental knowledge repositories at a Chinese dental school, spanning the years 2023 to 2025 (100 items per year). The 300 items were selected by the institutional examination committee from the Chinese Medical Education Question Bank—Oral Medicine Section (comprising approximately 84,000 items), a nationally standardized repository used in official graduation examinations. A stratified random sampling approach was applied by item type: 50% Type-I items (fundamental theoretical knowledge) and 50% Type-II items (clinical application and reasoning), reflecting the balanced competency framework of the dental program. This framework aligns with nationally validated competency assessment systems for professional dental degrees in China, which integrate formative and summative evaluations across basic science and clinical domains through expert consensus processes (23). Following initial stratified selection, an expert panel annually reviewed the 100-item sets to verify stable difficulty consistency and content validity across the 2023–2025 examinations, ensuring comparable item quality and cognitive demand year-to-year. While detailed psychometric indices are retained by the examination office and were not available for this retrospective analysis, the committee-documented stratified sampling by item type and annual expert calibration provide assurance of consistent item quality and balanced domain coverage across the three-year period.

To assess the models’ depth of understanding across the dental curriculum, items were categorized into two primary domains based on their content and cognitive demands:

(1) Basic science questions, assessing foundational knowledge in disciplines such as anatomy, physiology, microbiology, pathology, epidemiology, dental materials, orthodontics, and prosthodontics.
(2) Clinical reasoning questions, requiring application of diagnostic and treatment principles in case analysis, treatment planning, surgical strategies, and real-world decision-making.

### 2.3 Human reference data for performance benchmarking

To provide a human performance benchmark derived from an academically homogeneous cohort under identical examination conditions, anonymized reference data were obtained from a cohort of 93 undergraduate dental students who completed these assessments as part of their final-year curriculum. This cohort represented the full enrollment for each academic year (*n* = 29 in 2023; *n* = 32 in 2024 and 2025). All participants were fifth-year students in the same dental program, taught by the same faculty members following an identical syllabus. Assessments were conducted under identical standardized conditions and time constraints, with a total examination duration of 2 h for each annual cohort. Furthermore, all students were native Chinese speakers, ensuring a linguistically and academically homogeneous reference for comparing AI-driven knowledge alignment assessment against human clinical competency.

It is important to note fundamental differences between the human and AI testing conditions. Students completed examinations under authentic high-stakes conditions with strict 2-hour time constraints, cognitive stress, and no access to external resources. By contrast, LLMs were evaluated without time pressure, emotional burden, or attentional limitations, and were permitted multiple independent trials. These discrepancies mean that numerical score comparisons should be interpreted as reference-framed rather than equivalence-based: they indicate model-examination alignment rather than superior clinical capability.

### 2.4 Knowledge assessment protocol

The 300 questions were divided into three year-specific datasets (100 items per year). Each dataset was submitted to all three LLMs via their public web interfaces using a minimal-prompt, zero-shot protocol to emulate naturalistic examination conditions. No system role instructions, few-shot examples, chain-of-thought scaffolding, or task-specific prompting were provided. Questions were entered sequentially in their original Chinese text, followed only by a neutral instruction to provide answers in order (“请按顺序给出此份试卷题目的答案”). No additional context, examples, or system instructions were provided.

To obtain three independent trials per model, each run was conducted in a fresh user session to prevent carry-over of conversation history or contextual memory. All trials were performed using the same registered user account, a DELL Inspiron 5498 laptop, and Microsoft Edge browser. DeepSeek-R1 evaluations were completed on June 6, 2025, with all 300 questions processed within a single day. GPT-4o and GPT-4.5 evaluations were completed on June 10, 2025, with all 300 questions for both models processed within the same day. The temperature parameter was maintained at each platform's default setting for chat interactions. This protocol intentionally avoids prompt-engineering artifacts that could inflate model performance, yielding a conservative baseline estimate of raw knowledge alignment.

### 2.5 Statistical framework for system evaluation

System reliability and accuracy were assessed using a combination of descriptive and inferential statistics. Accuracy was defined as the percentage of correct answers per trial, with final model reliability expressed as mean ± standard deviation (SD) across the three runs.

For inferential comparisons, accuracy was calculated as the percentage of correct responses per trial (i.e., per run), with each independent run treated as a technical replicate. Between-model accuracy differences within each academic year were examined using one-way ANOVA with model as the fixed factor and the three independent runs as replicate observations (*n* = 9 per year: 3 models × 3 runs), followed by Tukey's *post hoc* test. Domain-specific performance across the pooled three-year dataset was assessed using a two-way ANOVA with model type and question category (basic science and clinical reasoning) as fixed factors and the three independent runs as replicate observations (*n* = 18: 3 models × 2 categories × 3 runs). Residual diagnostics confirmed that ANOVA assumptions were not substantially violated; therefore, parametric analyses were retained.

While we recognize that examination items within the same year share thematic clustering, our primary inferential goal was to compare model-level mean accuracy across years and domains. To ensure stable estimates while preserving statistical power, accuracy proportions were calculated per run within each domain stratum. For the year-specific one-way ANOVAs, each run-specific proportion represents the mean accuracy across 100 items per year. For the two-way ANOVA across knowledge domains, the basic science and clinical reasoning aggregates each represent the mean accuracy across 150 items (50 items/year × 3 years). This approach treats the independent run as the replicate unit of analysis rather than individual items, substantially reducing intra-cluster correlation concerns. We acknowledge that a mixed-effects model with random intercepts for examination year and/or item would represent a more statistically rigorous alternative for future studies with larger item banks. However, given the balanced design with independent runs as replicates and the primary focus on model-level differences, we believe the parametric framework provides valid directional inference.

To address concerns regarding item-level clustering, supplementary Friedman tests (nonparametric repeated-measures analysis) were conducted treating each examination item as a matched block. This approach does not assume independence among items or normality of residuals. The Friedman test confirmed significant overall model differences (*χ*^2^ = 81.34, *p* < 0.0001), with *post-hoc* comparisons directionally consistent with the parametric ANOVA framework (DeepSeek-R1 > GPT-4o, GPT-4.5 > GPT-4o, DeepSeek-R1≈GPT-4.5). These nonparametric robustness checks support the reliability of the reported model rankings despite the clustering limitation.

Intra-model response consistency was quantified based on the degree of agreement across the three independent runs for each question: a score of 1.0 was assigned when all three outputs matched exactly; 0.67 when two of the three outputs matched; and 0 when all three outputs differed. These agreement scores were then analyzed using a 3 × 2 chi-square test, with Bonferroni-adjusted pairwise comparisons. Statistical significance was set at *p* < .05.

All analyses were conducted using GraphPad Prism version 10.5.0 (GraphPad Software, San Diego, CA, USA).

## 3 Results

### 3.1 Benchmarking accuracy across annual standardized datasets

**Figure 1:**
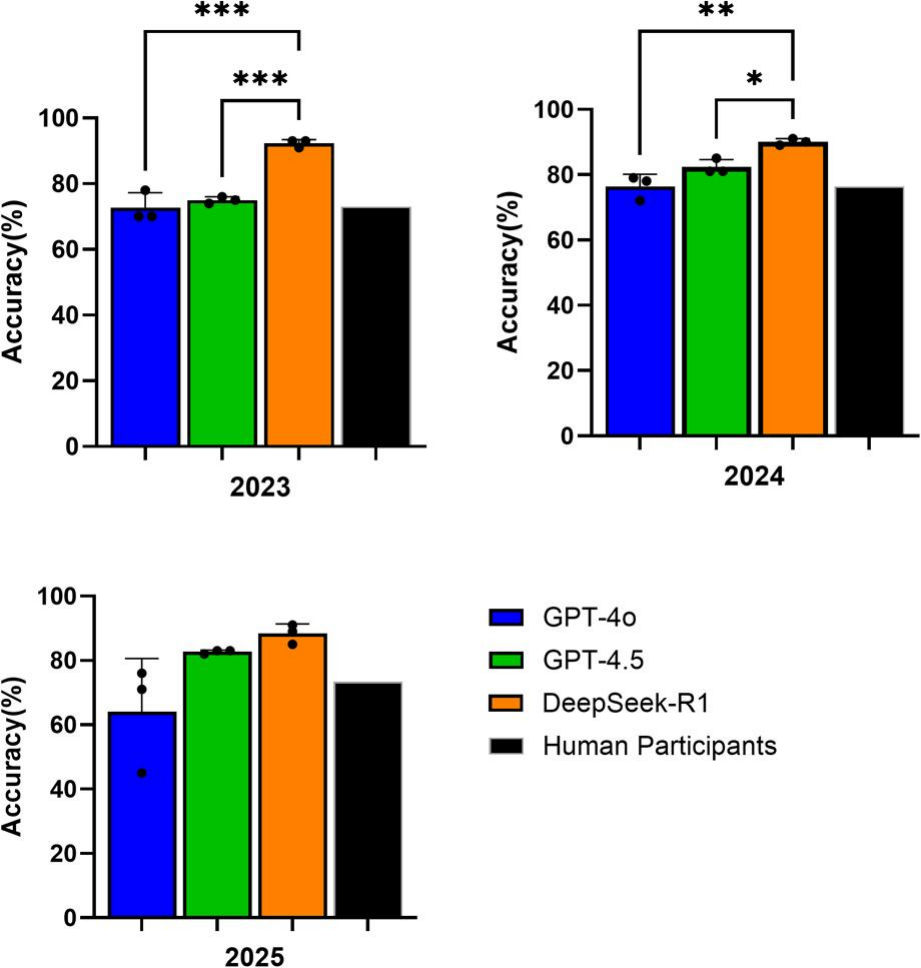
Mean accuracy (%) of GPT-4o, GPT-4.5, and DeepSeek-R1 on standardized dental graduation examinations from the Chinese dental school (2023–2025). Bars represent the means of three independent trials per model; error bars indicate the standard deviation. Human participant data reflect cohort sizes of *n* = 29 (2023), *n* = 32 (2024), and *n* = 32 (2025). Between-model comparisons were analyzed using one-way ANOVA followed by Tukey's *post hoc* test (*p < .05, **p < .01, ***p < .001).

The reasoning accuracy of GPT-4o, GPT-4.5, and DeepSeek-R1 was systematically evaluated using standardized dental competency datasets from 2023 to 2025 (Table 1, Figure 1).

**Table 1:** Accuracy of GPT-4o, GPT-4.5, DeepSeek-R1, and human participants on dental graduation examinations across academic years. Values are presented as mean accuracy (%). Between-model differences were assessed by one-way ANOVA with Tukey's *post hoc* test (p values shown for pairwise comparisons).

Academic Year		Number of questions		Mean accuracy(%)		p value		
		GPT-4o	GPT-4.5	DeepSeek-R1	Human Participants	DeepSeek-R1 vs. GPT-4o	DeepSeek-R1 vs. GPT-4.5	GPT-4o vs. GPT-4.5
2023	100	72.67%	75.00%	92.33%	73.01%	0.0003	0.0007	0.5937
2024	100	76.33%	82.33%	90.00%	76.41%	0.0017	0.0271	0.0698
2025	100	64.00%	82.67%	88.33%	73.34%	0.0512	0.7669	0.1254

In 2023, DeepSeek-R1 achieved 92.33% accuracy and significantly outperformed both GPT-4o (72.67%, *p* < .001) and GPT-4.5 (75%, *p* < .001), while GPT-4.5 and GPT-4o did not differ significantly (*p* = 0.5937). DeepSeek-R1 also surpassed the average human score for that year (73.01%), with GPT-4.5 performing slightly above and GPT-4o remaining close to the human benchmark.

In 2024, both DeepSeek-R1 (90.00%) and GPT-4.5 (82.33%) again outperformed the average human score (76.41%), while GPT-4o (76.33%) fell marginally below this benchmark. DeepSeek-R1 maintained the highest accuracy, and its advantage over both GPT models remained statistically significant (*p* < .05).

On the 2025 examination, DeepSeek-R1 (88.33%) and GPT-4.5 (82.67%) continued to outperform the human average (73.34%), whereas GPT-4o recorded its lowest accuracy (64.00%). No significant difference was observed between DeepSeek-R1 and GPT-4.5 in 2025 (*p* = 0.77), indicating comparable performance in that year.

Taken together, DeepSeek-R1 achieved the highest accuracy across all three years, peaking at 92.33% in 2023. By contrast, GPT-4o exhibited more variable and consistently lower performance.

### 3.2 Domain-Specific knowledge retrieval accuracy

**Figure 2:**
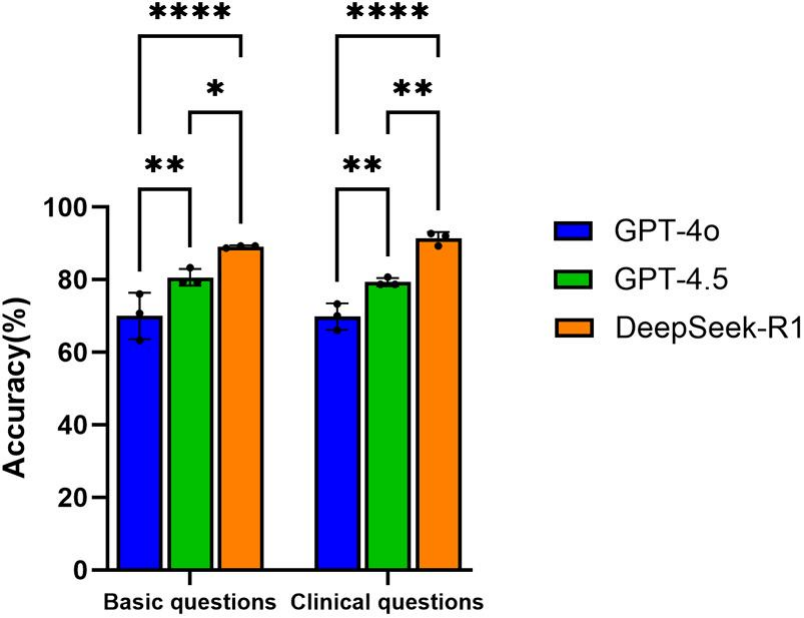
Mean accuracy (%) of GPT-4o, GPT-4.5, and DeepSeek-R1 on basic science and clinical reasoning questions (2023–2025). Statistical comparisons were performed using two-way ANOVA with model and question category (basic science and clinical reasoning) as fixed factors, followed by Tukey's *post hoc* test. (*p < .05, **p < .01, ***p < .001, ****p < .0001).

We next evaluated model performance across question categories, classifying items into basic science and clinical reasoning domains (Figure 2, Table 2). As in the year-wise analysis, DeepSeek-R1 achieved the highest accuracy in both domains (89.11% for basic science and 91.33% for clinical reasoning). GPT-4.5 followed with 80.66% and 79.34%, respectively. GPT-4o consistently performed least well across both categories, with scores of 70.00% for basic science and 69.78% for clinical reasoning.

**Table 2:** Accuracy of GPT-4o, GPT-4.5, and DeepSeek-R1 on basic science and clinical reasoning questions across all academic years. Values are presented as mean accuracy (%). Between-model differences were assessed by two-way ANOVA with Tukey's *post hoc* test(p values for pairwise comparisons are shown).

Question type	Number of questions	Mean accuracy (%)			p value		
		GPT-4o	GPT-4.5	DeepSeek-R1	DeepSeek-R1 vs. GPT-4o	DeepSeek-R1 vs. GPT-4.5	GPT-4o vs. GPT-4.5
Basic questions	150	70.00%	80.66%	89.11%	<0.0001	0.0204	0.0046
Clinical questions	150	69.78%	79.34%	91.33%	<0.0001	0.0019	0.0096

Two-way ANOVA results (Supplementary Table S1) indicated a significant main effect of model type on accuracy [F(2,12) = 58.26, *p* < 0.001], accounting for 90.00% of the total variance. By contrast, neither question type [F(1,12) = 0.0213, *p* = 0.886] nor the interaction between model and question category [F(2,12) = 0.465, *p* = 0.639] showed significant effects.

### 3.3 Output stability and reasoning reproducibility

**Figure 3:**
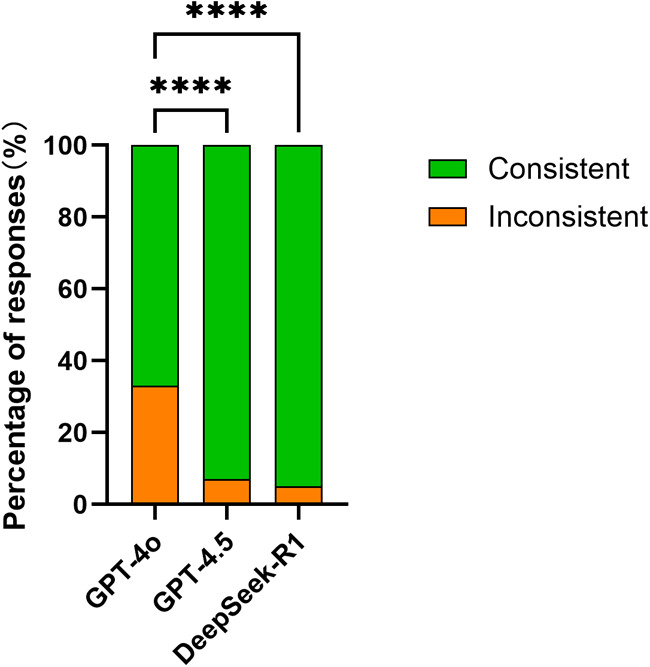
Proportion of consistent and inconsistent responses generated by GPT-4o, GPT-4.5, and DeepSeek-R1 across three independent runs (*n* = 300 questions per model). Bars represent 100% stacked distributions. Between-model differences in response consistency were assessed using *χ*^2^ tests with Bonferroni-adjusted pairwise comparisons (****p < .0001).

We finally evaluated the reproducibility of responses across three independent runs per model on the full 300-question dataset. DeepSeek-R1 demonstrated the highest agreement rate, followed by GPT-4.5, while GPT-4o showed substantially lower consistency across runs (Figure 3).

Bonferroni-adjusted pairwise comparisons confirmed that both DeepSeek-R1 and GPT-4.5 were significantly more stable than GPT-4o (adjusted *p* < 0.0001 for each comparison with GPT-4o). By contrast, the difference in agreement rates between DeepSeek-R1 and GPT-4.5 was not significant (adjusted *p* = 0.7673), indicating similar levels of response reproducibility. Together, these results suggest that DeepSeek-R1 and GPT-4.5 provide more consistent outputs when evaluated multiple times under identical conditions, supporting their robustness for high-stakes educational applications.

## 4 Discussion

### 4.1 **LLMs** performance as an indicator of standardized knowledge alignment

Although several studies have evaluated LLM performance on non-English dental and medical licensing examinations (6, 16, 18), few have benchmarked these models against real student cohort performance under identical institutional examination conditions. This study demonstrates that advanced LLMs, particularly DeepSeek-R1, achieve high accuracy on standardized dental examination items, indicating strong alignment between model outputs and the diagnostic principles embedded within formal dental curricula. The observed accuracy exceeding 90% reflects concordance with codified examination criteria rather than epistemological validation of curricular knowledge. It is essential to emphasize that the comparison between LLM accuracy and human student performance is reference-framed rather than equivalence-based. The human cohort completed examinations under authentic high-stakes conditions, including strict 2-hour time constraints, examination fatigue, and cognitive stress associated with graduation assessment. By contrast, LLMs operated without temporal pressure, emotional arousal, or attentional limitations, with model performance reflecting the mean of three independent runs under optimized conditions.

Therefore, numerically higher LLM accuracy should not be interpreted as superior clinical intelligence; instead, it indicates stronger alignment with codified answer keys under low-stress, repeated-sampling conditions. This distinction is critical: LLMs in this context serve as benchmarking references for examining item quality and curricular alignment, not as surrogates for human clinical competency. Nonetheless, this alignment-detection capacity may support future efforts to standardize and calibrate educational assessment materials within preventive dental training programs (24).

### 4.2 Model architecture as the primary determinant of educational reliability

The superior performance of DeepSeek-R1 appears attributable to both architectural design and alignment with Chinese clinical terminology (14, 16, 25). However, its advantage extends beyond linguistic adaptation. International benchmarking studies have reported comparable diagnostic reasoning performance on standardized medical licensing datasets (3), suggesting that reinforcement-learning–based architectures enhance alignment between internal model representations and structured clinical knowledge. Our two-way ANOVA findings further support this interpretation: model type, rather than question category, accounted for nearly all observed variance in accuracy. This indicates that educational reliability in LLMs is primarily governed by architectural properties rather than domain specificity.

### 4.3 Reasoning stability as a prerequisite for educational integration

For AI systems to be incorporated into educational infrastructures, output stability is as critical as accuracy. Inconsistent responses undermine trust and limit practical deployment in high-stakes instructional contexts. Our reproducibility analysis shows that DeepSeek-R1 and GPT-4.5 provide highly consistent outputs across repeated trials, whereas GPT-4o demonstrates substantially lower stability. This finding emphasizes that reasoning-optimized models are essential when LLMs are used as standardized knowledge alignment tools rather than exploratory chat systems (22, 26–29).

### 4.4 Potential future directions for preventive dental education applications

From a public health perspective, the capacity of LLMs to demonstrate alignment with complex clinical knowledge criteria may inform future exploration of how such tools could contribute to preventive dental education systems. However, it is important to emphasize that the present study does not directly evaluate educational outcomes, student engagement, or curriculum effectiveness; rather, it provides performance benchmarking data that may serve as an empirical reference for subsequent intervention studies. Effective prevention relies fundamentally on a workforce trained with accurate diagnostic principles. These preliminary findings raise the hypothesis that optimized LLMs might eventually contribute to intelligent tutoring systems and automated feedback modules, pending direct educational intervention trials to verify whether such alignment translates into measurable learning gains. Furthermore, emerging evidence from independent studies suggests potential for LLM application in automated essay scoring, extending theoretical utility beyond multiple-choice assessments (30)， though this capability was not evaluated in the present benchmarking study. Beyond student-facing tools, these models may provide a preliminary framework for hypothesizing about curriculum alignment and the pre-testing of institutional assessment items, potentially assisting educators in identifying areas where training materials may diverge from standardized curricular expectations, subject to prospective validation studies (6, 16, 18).

These findings carry several preliminary implications for dental educators. First, the high alignment observed for DeepSeek-R1 and GPT-4.5 suggests these models may serve as screening tools for identifying potentially problematic examination items—such as those with ambiguous wording or outdated content—prior to operational use. Second, the superior reproducibility of DeepSeek-R1 and GPT-4.5 highlights the importance of output stability in high-stakes educational contexts. Third, the comparable performance across basic science and clinical reasoning domains suggests that model architecture rather than domain specificity drives accuracy differences, which may inform procurement and deployment decisions. Recent evidence from LLM-as-grader studies in dental education further supports this cautious optimism. Hassanein et al. (31) reported that DeepSeek-3 achieved higher inter-rater reliability (ICC = 0.87) than ChatGPT-4 (ICC = 0.64) when grading clinical short-answer responses against human expert benchmarks, though both models exhibited systematic over-scoring of incorrect answers. This finding corroborates our observation of DeepSeek-R1's superior consistency and underscores the necessity of human-in-the-loop oversight even when LLMs demonstrate high alignment with institutional standards.

### 4.5 Contextual benchmarking, safety, and limitations

These findings underscore the necessity of localized benchmarking before implementing AI-driven educational technologies across healthcare education systems. Despite high accuracy, the known risk of hallucinations necessitates expert-supervised integration into educational workflows (32). Beyond hallucinations, broader fairness concerns warrant attention. Recent evidence indicates that LLMs may encode demographic biases that affect performance consistency across different clinical scenarios (33). In educational benchmarking, such latent biases could theoretically influence model accuracy on examination items involving underrepresented patient populations or rare disease presentations, underscoring the need for fairness auditing prior to any assessment deployment

Several boundary conditions limit the scope and interpretability of these findings. The scope of these findings is constrained by the study design. This evaluation was restricted to text-based, single-best-answer items from a single Chinese dental institution, examined within a single linguistic and curricular context. While this design enabled controlled comparison against a homogeneous student cohort, it precludes generalization to multimodal diagnostic environments, cross-institutional settings, or other languages. Recent studies have begun evaluating LLM vision capabilities in dental and maxillofacial contexts; for example, Nguyen et al. benchmarked large language model vision capabilities in oral and maxillofacial anatomy (34), and compared advanced reasoning vs. baseline models for histopathological diagnosis in oral and maxillofacial pathology (35). These investigations reveal both promising capabilities and persistent limitations in visual diagnostic accuracy, underscoring the substantial gap between our text-only benchmarking and real-world clinical practice. Furthermore, performance on standardized educational examinations reflects alignment with codified examination criteria rather than diagnostic capability in real-world patient care. Any extrapolation to clinical decision-support would require rigorous multimodal validation and regulatory oversight beyond the scope of this educational benchmarking study.

Methodological constraints further bound the interpretive confidence of these findings. The retrospective nature of the study precluded access to detailed item-level psychometric parameters (e.g., difficulty indices, discrimination coefficients) from the institutional examination office. While all items had undergone standard faculty validation before administrative use, the inability to report these metrics limits the depth of item quality assurance. Additionally, the statistical framework does not fully account for item-level clustering within examination years; a mixed-effects model with random intercepts for examination year would represent a more rigorous alternative for future studies with larger item banks. We addressed this limitation through supplementary nonparametric repeated-measures analyses (Friedman tests treating each examination item as a matched block), which confirmed significant overall model differences (*χ*^2^ = 81.34, *p* < 0.0001) and yielded directional conclusions consistent with the parametric ANOVA. The categorical agreement metric for consistency analysis (1.0, 0.67, 0) also represents a conservative approach that does not capture semantic equivalence, though it aligns with the binary correctness framework of formal examinations. Finally, web-based evaluation introduces uncontrolled backend variability (platform updates, undisclosed parameter adjustments), constraining strict reproducibility compared to API-based protocols with fixed parameters.

A third set of constraints pertains to the inherent epistemological limits of LLM benchmarking. A critical limitation across all contemporary LLM evaluations is the inability to fully exclude training data contamination. Although all 300 items predated the publicly disclosed training cutoffs of the evaluated models, structurally similar examination content or preparatory materials may exist within model training corpora—particularly widely circulated Chinese dental examination resources. Consequently, observed high accuracy may partially reflect exposure to semantically similar content during pre-training rather than *de novo* clinical reasoning.

We explicitly frame our contribution as localized benchmarking evidence within these bounded conditions, rather than universal claims regarding LLM utility in dental education. Future studies should prioritize multimodal evaluation incorporating radiographs and histological images; cross-institutional replication across diverse educational systems; prospective access to psychometric metadata; API-based protocols with fixed parameters; and adversarial testing with novel, never-published items to disentangle memorization from genuine reasoning.

## Data Availability

The data analyzed in this study is subject to the following licenses/restrictions: The datasets presented in this article are not readily available due to institutional regulations regarding the confidentiality of examination materials. Requests to access the datasets should be directed to the corresponding author, subject to institutional approval. Requests to access these datasets should be directed to He Meng, menghe@szu.edu.cn.
