# Supplementary material for: Large language models as data-driven engines for benchmarking preventive and clinical knowledge in Chinese dental examinations
Source: Front Oral Health. 2026 Jun 19;7:1849721. doi: 10.3389/froh.2026.1849721 (PMC13328340; doi:10.3389/froh.2026.1849721)
Supplement: Supplementary file 1 [file Table1.docx]

**Supplementary Material 1: Prompt Protocol**

The following is the complete text entered into each LLM interface during evaluation. No additional system instructions, role assignments, or few-shot examples were used.

User input (original Chinese): 请按顺序给出此份试卷题目的答案

[Followed by sequential pasting of examination items 1 through 100 for each year-specific dataset]

**Note:** This minimal-prompt approach was chosen to mirror authentic examination conditions in which students receive questions without scaffolding. We acknowledge that this protocol introduces greater response variability than engineered prompting frameworks; however, it provides a conservative baseline for benchmarking raw model knowledge retrieval.

**Supplementary Material 2：**

**Table S1. Two-way ANOVA results for accuracy of AI models across question types**

| **Source of Variation** | **SS** | **df** | **MS** | **F-value** | **p-value** | **%Total Variation** |
| --- | --- | --- | --- | --- | --- | --- |
| Model (Row) | 1240 | 2 | 620.2 | F(2,12)=58.26 | <0.0001 | 90.00 |
| Question Type (Column) | 0.2267 | 1 | 0.2267 | F(1,12)=0.0213 | 0.8864 | 0.02 |
| Interaction | 9.903 | 2 | 4.952 | F(2,12)=0.465 | 0.6389 | 0.72 |
| Residual | 127.7 | 12 | 10.65 | - | - | 9.27 |
| Total | 1377.8 | 17 | - | - | - | 100 |

SS : Sum of squares df : Degrees of freedom MS : Mean square

**Supplementary Material 3：Nonparametric Robustness Checks for Model-Level Accuracy Comparisons**

**Purpose:** The primary inferential analyses reported in the main text employed parametric ANOVA applied to accuracy proportions aggregated at the model × year level (n = 9 for one-way ANOVA; n = 18 for two-way ANOVA). To evaluate the robustness of the reported model rankings under less restrictive distributional and independence assumptions, we conducted supplementary nonparametric repeated-measures analyses.

**Statistical Methods:** Friedman tests were applied to the full item-level dataset (n = 300 examination items), with each item treated as a matched block and the three LLMs (DeepSeek-R1, GPT-4o, GPT-4.5) as the repeated-measures conditions. This nonparametric approach evaluates whether the distributions of accuracy rankings differ across models without assuming normality of residuals or independence among items within the same examination year. Following a significant Friedman test, Dunn's post-hoc comparisons with multiplicity adjustment were performed to identify pairwise differences between models.

Results. The Friedman test confirmed significant overall differences in accuracy rankings across the three models (χ² = 81.34, df = 2, p < 0.0001). Dunn's multiple comparisons with adjusted P values yielded the following pairwise results:

**Table S2. Dunn's Post-hoc Comparisons Following Friedman Test for Model-Level Accuracy Rankings**

| **Comparison** | **Rank Sum Difference** | **Adjusted P Value** | **Significant** |
| --- | --- | --- | --- |
| DeepSeek-R1 vs. GPT-4o | 129.0 | <0.0001 | Yes |
| DeepSeek-R1 vs. GPT-4.5 | 57.00 | 0.0599 | No |
| GPT-4o vs. GPT-4.5 | -72.00 | 0.0099 | Yes |

**Consistency with Parametric Analyses:** These nonparametric robustness checks yield directional conclusions consistent with the primary parametric ANOVA framework reported in the main text: DeepSeek-R1 and GPT-4.5 both demonstrate significantly higher accuracy than GPT-4o, while the comparison between DeepSeek-R1 and GPT-4.5 does not reach significance at the α = 0.05 threshold. The congruence between parametric and nonparametric approaches supports the reliability of the reported model rankings despite the acknowledged item-level clustering limitation.

**Data Structure:** The analysis utilized 300 matched blocks, corresponding to the 300 single-best-answer examination items evaluated across all three models. Within each block, the dependent variable was the mean accuracy across three independent runs per model (range: 0, 0.33, 0.67, 1.00).
